# Resting human trabecular meshwork cells experience tonic cation influx

**DOI:** 10.21203/rs.3.rs-4980372/v1

**Published:** 2024-08-28

**Authors:** Oleg Yarishkin, Monika Lakk, Christopher N. Rudzitis, Denisa Kirdajova, David Krizaj

**Affiliations:** University of Utah; University of Utah; University of Utah; University of Utah; University of Utah

**Keywords:** Ion channel, trabecular meshwork, calcium signaling, TRPC1, glaucoma, membrane potential

## Abstract

The trabecular meshwork (TM) regulates intraocular pressure (IOP) by converting biochemical and biomechanical stimuli into intracellular signals. Recent electrophysiological studies demonstrated that this process is mediated by pressure sensing ion channels in the TM plasma membrane while the molecular and functional properties of channels that underpin ionic homeostasis in resting cells remain largely unknown. Here, we demonstrate that the TM resting potential is subserved by a powerful cationic conductance that disappears following Na^+^ removal and substitution with choline or NMDG^+^. Its insensitivity to TTX, verapamil, phenamil methanesulfonate and amiloride indicates it does not involve voltage-operated Na^+^, Ca2^+^ and epithelial Na+ (ENaC) channels or Na^+^/H^+^ exchange while a modest hyperpolarization induced by SEA-0440 indicates residual contribution from reversed Na^+^/Ca2^+^ exchange. Tonic cationic influx was inhibited by Gd^3+^ and Ruthenium Red but not GsMTx4, indicating involvement of TRP-like but not Piezo channels. Transcriptional analysis detected expression of most TRP genes, with the canonical transcriptome pool dominated by TRPC1 followed by the expression ofTRPV1, TRPC3 and TRPC5. TRPC3 antagonist Pyr3 and TRPC1,4,5 antagonist Pico1,4,5 did not affect the standing current, whereas the TRPC blocker SKF96365 promoted rather than suppressed, Na^+^ influx. TM cells thus maintain the resting membrane potential, control Na^+^ homeostasis, and balance K^+^ efflux through a novel constitutive monovalent cation leak current with properties not unlike those of TRP channels. Yet to be identified at the molecular level, this novel channel sets the homeostatic steady-state and controls the magnitude of pressure-induced transmembrane signals.

## 1. Introduction

Trabecular meshwork (TM) cells are phagocytic and contractile, embedded in a multilayered ECM architecture that serves as a biological filter and resistor system for aqueous humor (Stamer and Clark, 2014; Mao 2024). Similar to smooth muscle cells that provide structural integrity for blood vessels and regulate vascular tone, TM cells physically support the vascular-lymphatic cells that form the canal of Schlemm and control the drainage of aqueous humor (Lutjen-Drecoll, 1999; Stamer and Clark, 2017). The cells are highly mechanosensitive, responding to changes in pressure, strain, shear and osmotic gradients with changes in ionic flux which modulate the cells’ shape, gene expression, contractility, and secretion of extracellular matrix (ECM) (Krizaj, 2020; Baumann et al., 2024; Mao, 2024; Redmon et al., 2024). Mechanosensitive membrane signaling is subserved by integrin-based cell-ECM contacts and mechanosensing TRPV4, Piezo1 and TREK-1 channels (Krizaj, 2020; Yarishkin et al., 2018; 2021; Zhu et al., 2021; Faralli et al., 2022) that control facilitatory and suppressive effects on trabecular outflow resistance and fibrotic remodeling (Krizaj, 2020; Lakk and Krizaj, 2021; Yarishkin et al., 2021; Zhu et al., 2021) while ion channels that subserve the resting TM membrane remains poorly understood.

Human TM cells are highly responsive to mechanical inputs, responding to physiological stimuli (strain, shear, swelling, pressure, indentation) with a current that reverses near the resting potential (V_rest_ ~−30 to −40 mV) (Yarishkin et al., 2018). V_rest_ itself is set by extra- and intracellular concentrations of permeant Na^+^, K^+^, Ca^2+^ and Cl^−^ ions and the relative expression of ion channels and transporters within the TM plasma membrane. Indicating the important function of the ionic mechanisms that maintain Vrest, the TM sensitivity to pressure is likely to be affected by shifts of the membrane potential into depolarizing or hyperpolarizing directions. TM to Ca^2+^ permeability modulates cytoskeletal dynamics, gene expression, secretion of ECM proteins and activity of Ca^2+^-dependent TRPM4 and BK channels (Wiederholt et al., 2000; Ryskamp et al., 2016; Lakk and Krizaj, 2021), Cl^−^ efflux, subserved by volume-regulated anion VRAC (LTTRC), CLC2 and/or anoctamine ANO6 channels contributes to cell volume regulation (Grant et al., 2013; Gasull et al. 2019, Banerjee et al., 2016; Baumann et al., 2024) and the K^+^ leak component, maintained by TREK-1 and auxiliary contributions from inwardly rectifying, voltage-gated K^+^, Ca^2+^-activated and TASK1 channels (Yarishkin et al., 2018; 2019), subserves the hyperpolarizing component of V_rest_ while the identity and functional properties of the leak conductance that balances TREK-1 activity under resting conditions remain unknown. In the present study we employed microfluorimetry in combination with whole cell recording and transcriptomic analyses to characterize the ionic mechanism that underpins homeostatic ionic signaling in the resting TM cell.

## 2. Materials and Methods

### 2.1. Cell cultures and transfection.

Immortalized cells, isolated from the juxtacanalicular region of the human eye (hTM cells), were procured from ScienCell Research Laboratories (Carlsbad, CA), and primary cultures of TM (pTM) cells were dissected from 3 human donors with no history of eye disease (healthy donors: 76-year-old female, 76-year-old male, 78-year-old male; POAG donors two 80-year old males and a 77-year old female; Utah Lions Eye Bank) following the consensus recommendations (Keller *et al*., 2018), and following the standards set by the WMA Declaration of Helsinki and the Department of Health and Human Services Belmont Report. The phenotype was periodically validated by profiling for TM markers *AQP*, *TIMP3*, *MYOC* and *MGP*, and by dexamethasone-induced upregulation of myocilin expression, as described (Lakk et al., 2021; Baumann et al., 2024; Redmon et al., 2024). Passage 2–6 (or up to 4 in case of pTM) cells were seeded onto Collagen I-seeded coverslips and grown in Trabecular Meshwork Cell Medium (ScienCell, Catalog#6591) at 37°C and 5% CO_2_.

### 2.2. Reverse Transcription and Quantitative Real-Time Polymerase Chain Reaction (Q-RT-PCR)

Total RNA was isolated using Arcturus PicoPure RNA Isolation Kit according to the manufacturer instructions (Applied Biosystems). One microgram of total RNA was used for reverse transcription. First-strand cDNA synthesis and PCR amplification of cDNA were performed using qScript^™^ XLT cDNA Supermix (Quanta Biosciences). SYBRGreen based real-time PCR was performed using Apex qPCR GREEN Master Mix (Genesee Scientific). The experiments were performed as triplicates of at least three independent experiments and are expressed as a ~ fold change compared to the control. The C_T_ method (ΔΔC_T_) was used to measure relative gene expression where the ~ fold enrichment was calculated as: 2 - ^[*Δ*CT (sample) − *Δ*CT (calibrator)]^ after normalization. GAPDH and β-tubulin were utilized as endogenous controls to normalize the fluorescence. The primer sequences are listed in Table 1.

**Table:** 

Name	Forward primer	Reverse primer	NCBI reference number
TRPV1	GCCCAGCATGTTCCCAAATC	TGTCCCAGTAGAGACTGACCA	NM_080704.4
TRPV2	GAGGAGGTGAACTGGGCTTC	CTCGAGAGTTCGAGGGACAC	NM_016113.5
TRPV3	CCTTTTCTCCGGTGGGGATG	GCTTTCATGGCTGGTGAGGT	NM_001258205.2
TRPV4	TCCCATTCTTGCTGACCCAC	AGGGCTGTCTGACCTCGATA	NM_021625.4
TRPC1	TGCGTAGATGTGCTTGGGAG	CGTTCCATTAGTTTCTGACAACCG	NM_001251845.2
TRPC3	CTCTGGAGGTACACAGGCAC	CAGAATTTTCCCCAGCCTCG	NM_001130698.2
TRPC4	AAGATTGGGACAATGGGACC	ACGGTTTTGCTACTGGTGAA	NM_016179.4
TRPC5	GGGAGGGGCCAGAAATAGGA	ACAGTCTTAGCGAAGCAGGG	NM_012471.2
TRPC6	TTGTGCCATTTCTGGGAGCA	CCAGCATCCTCTGAATGCCAA	NM_004621.6
TRPC7	CCTCTAGGACACGAAGGGGA	GCTGGACCAAAGGTCTCACA	NM_020389.3
TRPM2	GACCTTCACGGAAAGCAGGA	ATCTGAAGGCTTCCACTGCC	NM_003307.3
TRPM4	GATGCACACCAGGGAGAA	AGAGCCGGAGGAAATTGCTG	NM_017636.4
TRPM8	ACACCTGTAGTCCCAGCTTTC	TTCCTCCTTCAGCCAGTGAG	NM_024080.5
TRPA1	TGGCTATGTATGTGTCCTTGTG	AGGCCCCGTACTAGATGGAA	NM_007332.3
TRPP2	CGCCGGGAAGAAAGGAACAT	CTGCATCTCGATCTCCAGGC	NM_000297.4
GAPDH	CTCCTGTTCGACAGTCAGCC	GACTCCGACCTTCACCTTCC	NM_002046.5
β-tubulin	CCTTCCTCCACTGGTACAC	TCTGCCTTAGGCCTCCTCTT	AF141349.1

### 2.3. Electrophysiology.

TM cells (passage 2 to 6) were plated on collagen type 1-coated glass coverslips 1–2 hrs prior to experiments and perfused with standard extracellular saline containing (in mM): 135 NaCl, 2.5 KCl, 1.5 MgCl_2_, 2 CaCl_2_, 10 HEPES, 5.6 D-glucose (pH 7.4). In some experiments, NaCl was substituted with equimolar concentrations of choline chloride, N-methyl-d-glucamine (NMDG) chloride, CsCl, or LiCl. Whole-cell currents were recorded at −40 mV, with the pipette solution containing (in mM): 135 K-gluconate, 10 KCl, 2 HEPES, 1 MgCl_2_, 10 ethylene glycol-bis(β-aminoethyl ether)-N,N,N’,N”-tetraacetic acid (EGTA) (pH 7.3). The whole-cell current and membrane potential were recorded at room temperature (20–22° C) with Multiclamp 700B amplifier, Clampex 10.7 acquisition software, and a Digidata 1550 digitizer (Molecular Devices), with data sampled at 5 kHz and filtered at 2 kHz with an 8-pole Bessel filter. Recording pipettes, pulled using P-2000 puller (Sutter Instruments), had a resistance of 5–8 MΩ when filled with the pipette solution. Data was analyzed with Clampfit 10.7 (Molecular Devices) and Origin 8 Pro (Origin Lab) software.

### 2.4. Calcium and sodium imaging.

The single excitation Na^+^ indicator NaTRIUM Green^™^-2 acetoxymethyl dye (3 μM; TEFLabs) was used for intracellular ion measurements (Yarishkin et al., 2022). The cells were loaded for 45 min at 37° C in a CO_2_/O_2_ cell culture incubator. Images were acquired on an inverted Nikon Ti microscope at 40x magnification and binned at 2×2 (Ryskamp et al., 2011). Cells from 3 separate pTM strains were seeded on glass coverslips for 3–6 hours prior to experiments. The cells were perfused with extracellular solution containing (mM): 135 NaCl, 2.5 KCl, 1.5 MgCl_2_, 2 CaCl_2_, 10 HEPES, 5.6 D-glucose (pH, 7.4, osmolarity, 300–303 mOsm) (Lakk et al., 2018; 2021), with a subset of experiments substituting extracellular NaCl with cholineCl. 490 nm excitation (Semrock) was delivered by a light guide from a Xenon arc lamp (DG4, Sutter Instruments), with emission collected above 510 nm captured with a Delta Evolve or Prime 95B EMCCDs (Photometrics). Backgrounds on each slide were subtracted with NIS Elements (Nikon). The imaging experiments were conducted at room temperature (20–22° C).

### 2.5. Reagents.

Reagents, including salts, 1-[2-(4-methoxyphenyl)-2-[3-(4-methoxyphenyl) propoxy]ethyl-1H-imidazole hydrochloride (SKF 96365), verapamil, amiloride and Pyr3 were purchased from Sigma, Cayman Chemicals or ThermoFisher. The *Grammostola spatulata* mechanotoxin GsMTx4 was from Alomone Labs and Pico 1,4,5 was from GLPBIO.

### 2.6. Data analysis.

Statistical comparisons were made with one-way ANOVA test followed by post-hoc Tukey’s multiple comparison of means or Student’s paired *t*-test or two-sample *t*-test (Origin 8.0, Origin Lab Corporation). Na^+^ imaging results were compared using repeated measures one-way ANOVA. A difference of P ≤ 0.05 (*), P ≤ 0.01 (**) and P ≤ 0.001 (***) were considered statistically significant. Results are presented as the mean ± S.E.M.

## 3. Results

### 3.1. TM cells possess a constitutively active cation influx pathway

**Figure 1:**
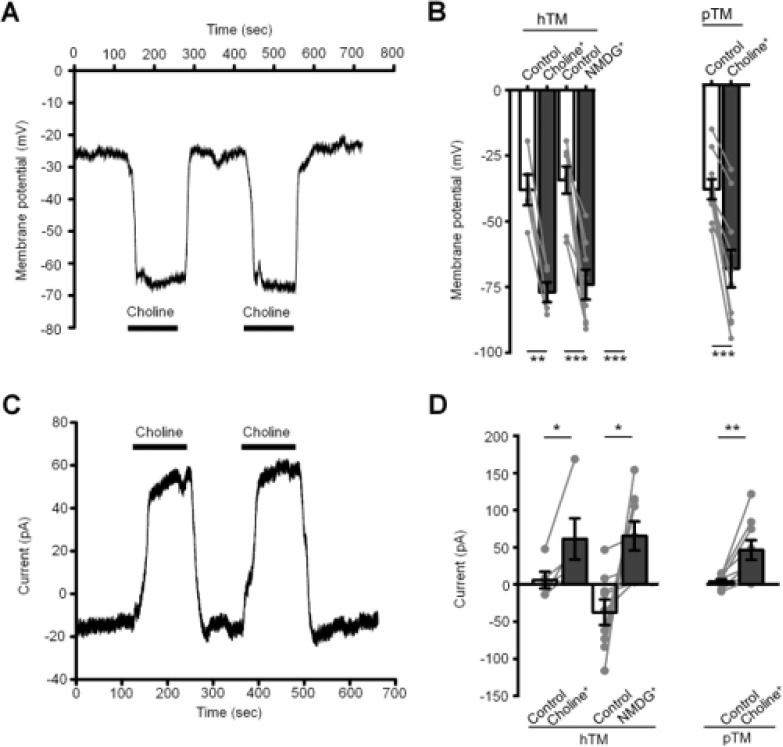
Constitutive cation influx in resting human TM cells. Whole cell recording, current-clamp. **(A)** Substitution of external Na^+^ with nonpermeable quaternary cations hyperpolarizes the TM resting potential and induces a positive shift in I_hold_. Choline substitutions result in rapid, reversible and reproducible hyperpolarizations. (B) Averaged data for [Na^+^]o substitution with external choline and NMDG. The response of pTM cells was identical to the hTM response (C & D) Voltage clamp. [Na^+^]o substitution with choline and NMDG is associated with a positive shift in the holding current, indicating loss of tonic cation influx. *, P <0.05; ** P<0.01; *** P < 0.005.

The resting membrane potential (V_rest_) of TM cells is ~ −40 mV, with its deviation from the Nernst potential for K^+^ (~ −100 mV), Na^+^ (~^+^61 mV), Ca^2+^ (^+^137 mV) and Cl^−^ (~−64 mV) ions indicating concurrent activation of multiple ion channels. We recently characterized the properties of the K^+^ channel that mediates the principal hyperpolarizing component of TM V_rest_ (Yarishkin et al., 2018, 2019) while the properties of the cationic channel that provides the depolarizing component remain unknown. An insight into its permeation properties was sought by substituting extracellular Na^+^ ([Na^+^]_o_) with non-permeant quaternary ammonium choline and N-methyl-d-glucamine (NMDG^+^), as well as Li^+^ or Cs^+^ ions. Choline and NMDG^+^ substitutions resulted in immediate shift in V_rest_, indicated by hyperpolarization from −30.4 ± 4.7 mV to −61.6 ± 3.0 mV (choline; n = 5 cells) and from −27.4 ± 4.1 mV to −59.3 ± 4.6 mV (NMDG; n = 8 cells) (Fig. 1). Reduced Na^+^ influx was associated with a positive shift in I_hold_ (holding current) from 5.9 ± 11.3 pA to 61.3 ± 27.7 pA (choline) and −37.4 ± 17.0 pA to 65.2 ± 19.4 pA (NMDG) (Fig. 1C & D). pTM cells displayed similar responsiveness to choline substitution (Fig. 1B), with a hyperpolarizing shift from −31.8 ± 3.0 mV to −56.0 ± 5.7 mV and a positive shift of the transmembrane current from 4.0 ± 3.1 pA to 46.3 ± 13.1 pA (Fig. 1D) (n = 10).

**Figure 2:**
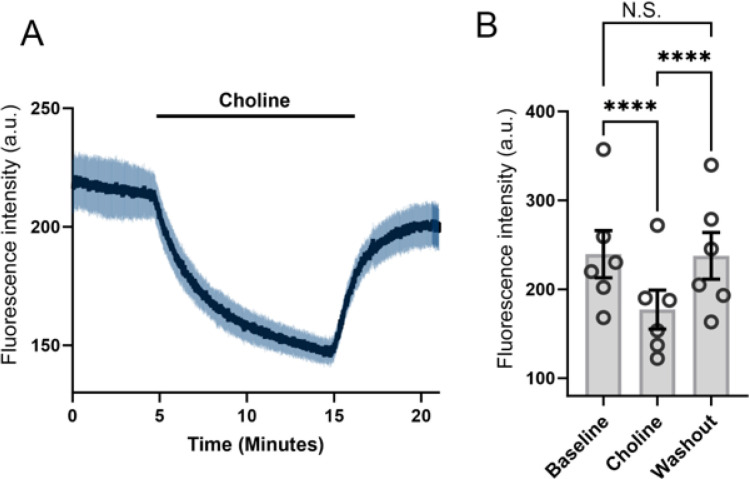
Na^+^ imaging. Constitutive Na^+^ influx is required to maintain steady-state [Na^+^]i. (A & B) [Na^+^]_o_ substitution with nonpermeable choline induces gradual reduction in [Na^+^]i. (B) Averaged data for the experiment shown in A. **** P < 0.001

We additionally tested the contribution of constitutive monovalent cation influx to steady-state [Na^+^]i with fluorescent imaging based on the membrane-permeable Na^+^-sensitive dye NaTRIUM Green^™^ (Yarishkin et al., 2022). Consistent with reduced tonic Na^+^ influx, [Na^+^]_o_ replacement with equimolar [choline]_o_ significantly reduced [Na^+^]_i_ (P < 0.001; n =34)(Fig. 2). The rate of [Na^+^]_i_ decline presumably reflects activity of endogenous Na^+^/K^+^ pumps. Tonic K^+^ efflux mediated by TREK-1 channels (Yarishkin et al., 2018; 2019) is thus balanced by a constitutively active Na^+^ influx pathway acting in parallel with Na^+^ clearance.

### Constitutive depolarizing influx is nonselective for monovalent cations

**Figure 3:**
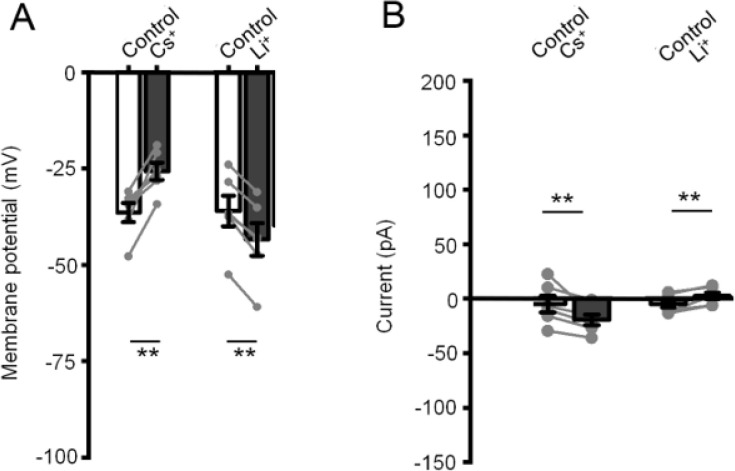
Whole cell recording. The constitutive influx pathway is permeable to monovalent cations. Whole cell recording. (A) Substitution of [Na^+^]_o_ with [Li^+^]_o_ and [Cs^+^]_o_ does not reproduce the dramatic effect of quaternary cations. Cs^+^-based saline induces a small but significant (P <0.01) depolarizing shift in contrast to Li^+^-based saline, which induces a small hyperpolarization (P < 0.01). (B) The two ions are associated with reduction and increase in the holding current (P < 0.01), respectively.

To ascertain whether the tonic cation influx mechanism discriminates between monovalent cations, [Na^+^]_o_ was substituted for [Cs^+^]_o_ and [Li^+^]_o_. The Cs^+^-based saline induced a modest but significant (P < 0.01) depolarization from −29.1 ± 1.2 mV to −20.6 ± 1.8 mV and a negative I_hold_ shift from −4.9 ± 7.6 pA to −19.4 ± 4.9 pA (Fig. 3) (n = 6) whereas Li^+^-based extracellular solution was associated with a hyperpolarization, from −28.8 ± 3.2 to −34.7 ± 3.4 mV (P < 0.01) and V_hold_ shift from −4.9 ± 3.4 pA to 2.3 ± pA (n = 6). Both cations are thus able to substitute for Na^+^ in maintaining V_rest_, with neither reproducing the dramatic effects of choline/NMDG substitutions. Their effect on V_rest_ might reflect differential channel permeability to monovalent cations with reduced permeability for cations having larger hydrated radius (Cs^+^ > Na^+^ > Li^+^).

### Constitutive Na^+^ influx is not mediated by ubiquitous transporters, exchangers or voltage-operated channels

**Figure 4:**
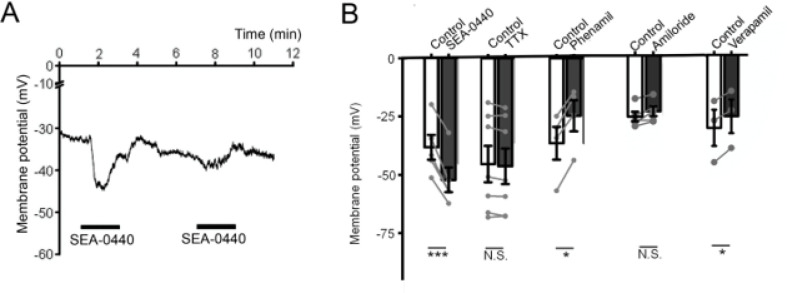
Na^+^/Ca^2+^ exchange modulates V_rest_ whereas Nav, Cav, ENaC, HCN channels and Na^+^/H^+^ exchange do not. (A) The NCX exchange antagonist SEA-0440 hyperpolarizes the cells in a use-dependent manner ((peak 1^st^ exposure, showing tachyphylaxis). (B) Averaged V_rest_ data in the presence of NCX (SEA-0440), voltage-gated Na^+^ channel (TTX), ENaC (phenamyl acetate), Na/H exchange and HCN (amiloride) and voltage-gated Ca2^+^ (verapamil) channels.

To ascertain the molecular identity of the mechanism mediating tonic influx of Na^+^, we evaluated the effects of obvious Na^+^ channel and electrogenic transporter candidates, such as degenerins/epithelial sodium channels (E_Na_C; Krueger et al., 2012), voltage-gated Na^+^ channels (Rich et al., 1999), Na^+^/H^+^ and Na^+^/Ca^2+^ exchange. TTX, an inhibitor of inactivating and noninactivating voltage-operated Na^+^ channels had no effect on rest at the concentration (1 μM) that antagonizes TTX-sensitive (TTX-s) and “resistant” (TTX-r) isoforms. Amiloride (3 μM), an inhibitor of Na^+^/H^+^ exchange and E_Na_Cs had no effect on V_rest_. Verapamil (10 μM), a broad-spectrum non-dihydropyridine inhibitor of voltage-gated Ca^2+^ channels and ATP-binding cassette (ABC) transporters (Collins et al., 2023) and phenamil methanesulfonate (3 μM), another inhibitor of DEG/E_Na_C channels, evoked modest depolarizations (Fig. 4B). The Na^+^/Ca^2+^ exchange inhibitor SEA-0440 hyperpolarized the cells by ~11 mV, but the tachyphylaxis associated with NCX inhibition argues against a central role in constitutive cation influx (Fig. 4A). The tonic pathway thus does not involve TTX-sensitive Na^+^ conductances, epithelial Na^+^ channels, Na^+^/H^+^ exchange and reversed NCX exchange.

### Constitutive Na^+^ influx may involve TRP channels

**Figure 5:**
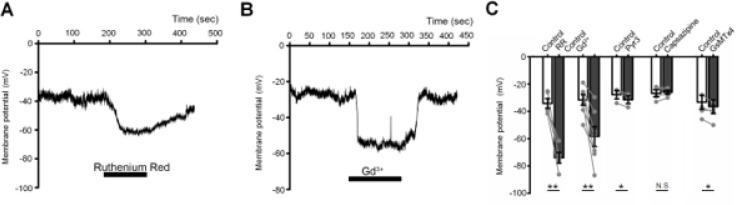
Nonselective TRP channel blockers suppress constitutive cation influx. Whole cell recording. (A & B) Superfusion with Ruthenium Red and Gd3+ was associated with persistent hyperpolarization (B) inhibition of TRPC3 (Pyr3), TRPV1 (capsazepine) and Piezo channels (GsMTx4) does not affect the standing current. * P<0.05; ** P<0.01

Piezo and TRP channels are widely expressed superfamilies of largely nonselective cation channels with many if not most cells in the vertebrate eye expressing Piezo 1/2 and isoforms across multiple TRP families (Gilliam and Wensel 2011; Lapajne et al., 2022; Krizaj et al., 2022; Yang et al., 2022). We tested the possibility that constitutive Na^+^ influx reflects resting activity of Piezo or TRP channels using two broad-spectrum TRP inhibitors, Ruthenium Red (10 μM) and Gd^3+^ (100 μM). Both induced prominent hyperpolarizations: from −34.2 ± 3.6 mV to −74.1 ± 4.0 mV (Ruthenium Red) and from −31.6 ± 3.9 mV to −58.4 ± 7.3 mV (Gd^3+^) (Fig. 5). The Piezo1 channel represents a major constituent of the nonselective cation transcriptome in TM cells (Yarishkin et al., 2021) yet the antagonist GsMTx4 (5 mM) slightly potentiated cationic influx (Fig. 5C). The nonselective cation conductance in TM cells thus involves a TRP-like not Piezo1-like channel, but not is not associated with TRPC3 or TRPV1 activation.

### Molecular expression of TRP channels in TM cells.

**Figure 6:**
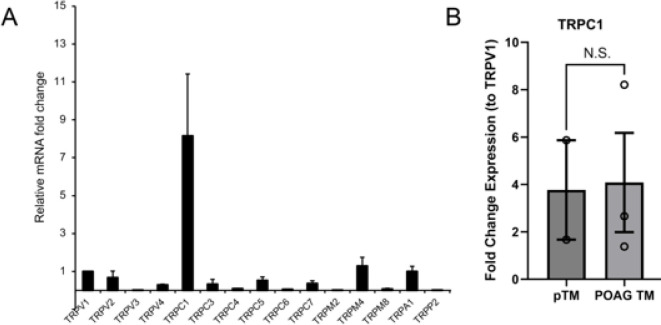
The expression pattern for principal TRP isoforms in pTM cells, normalized to TRPV1. (A) TRPC1 expression was the most abundant, followed by TRPM4, TRPA1 and TRPV1 mRNAs. (B) TRPC1 expression in pTM vs gTM cells (N = 3).

We profiled the TM transcriptome to identify TRP candidates for tonic cation influx. The expression profile was dominated by TRPC1 (Fig. 6A), followed by the expression of TRPM4>TRPA1>TRPV1>TRPV2>TRPC5>TRPV4 mRNAs. Expression of the TRPC1 gene in gTM samples derived from 3 different POAG donors was not different from healthy donor samples (Fig. 6B).

### The standing cationic influx is not mediated by TRPC1 homomers or heteromers

To assess the involvement of TRPC1 channels in maintaining the standing cationic inward current, we exposed the cells to SKF96365, a general inhibitor of TRPC channels (Szikra et al., 2009; Molnar et al., 2016). Unexpectedly, Na^+^ Green -loaded cells responded to the inhibitor (25 mM) with large increases in [Na^+^]i. While substituting [Na^+^]o with choline obviated this effect (n =3) to show it is mediated by a Na^+^-permeable channel, the SKF96365 -independence of tonic Na^+^ influx argues against the involvement of TRPC1 (and TRPC isoforms in general). Consistent with this, Pico-145 (5 mM), an antagonist of TRPC4, TRPC5 homomers and TRPC1/4,5 heteromeric channels (Rubaiy et al., 2017) did not induce obvious changes in TM membrane potential or current (n=3). The TRPC3 inhibitor pyrazole 3 (Pyr3; 5 mM), which does not affect other DAG-sensitive canonical isoforms but blocks Orai channels (Schleifer et al., 2012), had a modest facilitatory effect whereas the TRPV1 channel capsazepine (5 mM) (Jo et al., 2017) had no effect (Fig. 5C).

## Discussion

In this study we identify show that a novel monovalent cation leak pathway that maintains the resting membrane potential in unstimulated human TM cells. The constitutive cation influx mediated by the leak channels was largely unaffected by substituting Na^+^ with Li^+^ and Cs^+^, resisted Piezo1, epithelial Na^+^ and voltage-gated Ca^2+^ or Na^+^ channel inhibitors but was blocked by nonselective inhibitors of TRP channels. The new conductance, which remains to be identified at the molecular level, plays a fundamental function as a main regulator of the cells’ V_rest_ that balances mechanosensitive TREK-1 activity (Yarishkin et al., 2018), controls the driving forces for K^+^, Na^+^, Ca^2+^ and Cl^−^ fluxes, and modulates the amplitude of the pressure-activated current.

The rapid sustained hyperpolarization and [Na^+^]_i_ decrease observed following substitution of extracellular Na^+^ with choline or NMDG^+^, quaternary amines that cannot cross the plasma membrane or pass through Na^+^ channels due to their fixed charge, indicate the presence of constitutive cationic influx in hTM/pTM cells. Our observation that the depolarizing leak channel is permeable to Na^+^, Li^+^ and Cs^+^ indicates that it does not discriminate between small monovalent cations. Prior studies associated Na^+^ homeostasis in TM cells with E_Na_C channels (Krueger et al., 2012), Na^+^/H^+^ exchange (Stumpff and Wiederholt, 2000; Chu et al., 1992) and a voltage-dependent Na^+^ current (Rich et al., 1999). The insensitivity of V_rest_ to micromolar concentrations of TTX indicates no Na_v_ channel involvement. Because E_Na_C and Ca^2+^ channel inhibition should hyperpolarize the cells we thus cannot explain the small depolarizing effects of verapamil and phenamil methanesulfonate; more studies are required to understand these effects, which we tentatively ascribe to nonselective action. Amiloride insensitivity argues against involvement of E_Na_Cs and Na^+^/H^+^ exchange and excludes a major role for proton homeostasis (Yarishkin et al., 2019a), and insensitivity to amiloride and Cs^+^ excludes a role for the HCN current that might follow TREK-1 mediated tonic hyperpolarization (Yarishkin et al., 2018; Yarishkin et al., 2019a, b). The modest (~11 mV) yet consistent hyperpolarization induced by SEA-0440 is, however, of interest as indicates the possibility that resting TM cells experience calcium influx via reversed Na^+^/Ca^2+^ exchange.

Na^+^ substitution with Cs^+^ and Li^+^ induced modest (~ 5 mV) Vrest changes in opposite directions, depolarizing (Cs^+^) and hyperpolarizing (Li^+^), with neither replicating the powerful hyperpolarizations induced by nonpermeable cations. This indicates that the constitutive influx pathway is permeable to small monovalent cations, with the differential effects on V_rest_ and I_hold_ potentially reflecting the effects of different radii, differential effects on the Na^+^/K^+^ ATPase and/or Cs^+^/Li^+^-sensitive channels and transporters.

In contrast to the lack of the effect of voltage-gated channel and exchange inhibitors, the block of tonic influx by broad-spectrum TRP inhibitors Ruthenium Red and Gd^3+^ accords with potential involvement of TRP or other non-selective cation channels. Of the seven members (TRPC1–7) of the canonical family we found expression to be by far the most prominent for the TRPC1 gene, with additional TRPC3, TRPC5 and TRPC7 signals, as well as TRPM4, TRPA1, TRPV1, TRPV2 and TRPV4 expression. Previous PCR immunostaining experiments localized TRPC1, TRPC3, TRPC4, and TRPP2 expression to bovine and human TM (Abad et al., 2008; Tran et al., 2014). Apart from TRPV4, which has been implicated in regulation of TM contractility and conventional outflow (Luo et al., 2014; Ryskamp et al., 2016; Redmon et al., 2024) and TRPM4, a Na^+^ channel downstream from TRPV4 (Yarishkin et al., 2022), our understanding of the expression and function of the other 26 TRP isoforms is minimal for any mammalian TM preparation. The expanded roster of expressed TRP genes will hopefully encourage further study of these polymodal cation channels. In particular, we would like to highlight the potential significance of the TRPC1 channel, which dominates the pTM TRP transcriptomic profile. This vertebrate homolog of the *Drosophila* photochannel (Krizaj et al., 2023) encodes a protein that operates in receptor-operated and store-operated modes, with ubiquitous expression across ocular tissues, with unknown cellular functions in the eye (Szikra et al., 2009; Molnar et al., 2012; 2016) but altered expression pattern in retinitis pigmentosa (Caminos et al., 2023). TRPC1 interaction with Piezo1 and TRPV4 channels during transmission of traction forces (Budde et al., 2024) could be relevant within the context of trabecular outflow resistance (Yarishkin et al., 2019b; Krizaj, 2020) yet its expression in gTM cells was not significantly different from pTM controls. Studies in lactotrophs, pituitary cells and GH3 cells associated TRPC1-like channels with a background Na^+^ conductance (Kučka et al., 2012). Arguing against a major role for TRPC1 in mediating the constitutive influx, SKF96365, widely used to block TRPC channels in heterologously expressed cells and native systems (Okada et al 1998; Szikra et al., 2008; 2009; Molnar et al., 2016), increased [Na^+^]_i_ – an effect that cannot be explained by suppression of tonic influx, or inhibition of TRPC channels, NCX and SERCA pumps. TRPC1 does not form functional pores, requiring heteromerization with TRPC3, TRPC4, TRPC5, TRPC6, TRPV4, TRPP2 and/or Orai proteins (Strübing et al. 2001; Alfonso et al., 2008; Storch et al., 2012; Park et al., 2023) that we and others (Abad et al., 2008; Tran et al., 2014) find expressed within the TM. pTM cells express its heteromeric partners TRPC3 (Woo et al., 2014), TRPC4 (Park et al., 2023) and TRPC5 (Broker-Lai et al., 2017) (Fig. 6) but the V_rest_ insensitivity to Pyr3 and Pico 1,4,5 argues against involvement of TRPC3, TRPC4, TRPC5 homomers and TRPC1/3/4/5 heteromers. The second most prominently expressed transcript was TRPM4, encoding a Ca^2+^-activated Na^+^ channel that may play a role in oscillatory activity and cell swelling downstream from TRPV4 activation (Yarishkin et al., 2022; Baumann et al., 2024). Future studies may look into the involvement of NALCN, a Na^+^ leak channel that subserve non-selective voltage-independent leak currents in subsets of neurons, heart and secretory organs (Lu et al., 2007; Monteil et al., 2024). Similar to the tonic current in TM cells, NALCN is permeable to Cs^+^ and Li^+^, inhibited by replacing Na^+^ with NMDG^+^, insensitive to TTX, inhibited by Gd^3+^ and resistant to SKF96365 (Lutas et al., 2016; Monteil et al., 2024). NALCN but not the TM standing current, however, is inhibited by verapamil (Lu et al., 2007; Monteil et al., 2024).

In conclusion, we characterized the properties of a powerful novel ionic conductance in human TM cells with potentially significant functions in outflow regulation, fibrotic remodeling and glaucoma. From a physiological perspective, this constitutively activated channel controls the driving forces for Na^+^, K^+^ Cl− and Ca^2+^ ions and thus determines the reversal potential for the pressure-activated current. Following its identification, this channel could be targeted to regulate the magnitude of the TM pressure response mediated by Piezo1, TRPV4 and TREK-1 channels.

## Figures and Tables

**Figure 7:**
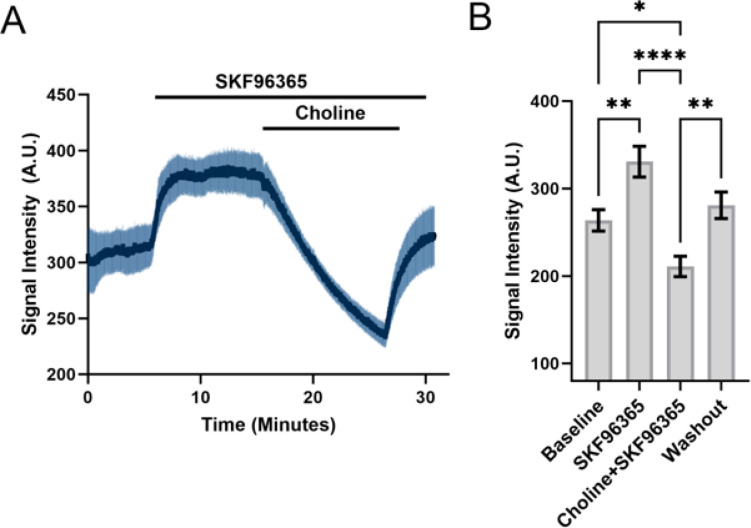
Na^+^ imaging. TRPC inhibitor SKF96365 does not antagonize constitutive cation influx. (A) Superfusion with SKF96365 was associated with an increase in Na-Green fluorescence indicating activation of a cytosolic Na^+^ influx pathway; obviation of the response following Na^+^ substitution with choline indicates that the increase is mediated by Na^+^ influx. (B) Data summary for the experiment shown in A.
